# Long non-coding RNA SOX21-AS1 modulates lung cancer progress upon microRNA miR-24-3p/PIM2 axis

**DOI:** 10.1080/21655979.2021.1955578

**Published:** 2021-09-13

**Authors:** Fengfeng Wang, Tengfei Gu, Yao Chen, Yu Chen, Dan Xiong, Yehan Zhu

**Affiliations:** a Department of Pulmonary and Critical Care Medicine, The First Affiliated Hospital of Soochow University, Suzhou, China; b Department of Respiration, People’s Hospital of Jingjiang, Jingjiang, Jiangsu, China; c Department of Anesthesiology, People’s Hospital of Lianshui County, Lianshui, Jiangsu, China

**Keywords:** Lung cancer, SOX21-AS1, miR-24-3p, PIM2

## Abstract

Lung cancer is a lethal cancer that threatens human health. Several studies have demonstrated the role of long non-coding RNAs (lncRNAs) in lung cancer. *SOX21-AS1* is a newly discovered oncogenic lncRNA, but its molecular mechanism in lung cancer is not known. Here, the levels of *SOX21-AS1, miR-24-3p*, and PIM2 were examined in lung cancer and normal tissues. The relationships between *miR-24-3p* and *SOX21-AS1* or PIM2 were predicted using bioinformatics tools and confirmed using a luciferase reporter assays. Colony formation, MTT, flow cytometry, and transwell assays were conducted to analyze cell proliferation, apoptosis, migration, and invasion abilities, respectively. Western blotting was used to measure PIM2 expression levels in cancer tissues and cells. *SOX21-AS1* expression levels were high in lung cancer tissues and cells. In contrast, the amount of *miR-24-3p* bound to *SOX21-AS1* was relatively low in cancerous tissues and cells. The knockdown of *SOX21-AS1* decreased cell proliferation, activated apoptosis, and promoted cell migration and invasion. These effects were abolished by *miR-24-3p* inhibition. The oncogenic function of *SOX21-AS1* mediated through targeting *miR-24-3p* was also demonstrated in animal models. PIM2 was targeted by *miR-24-3p* and showed increased levels in tumor tissues and cells. Furthermore, *miR-24-3p* overexpression inhibited the proliferation and promoted the apoptosis of lung cancer cells. In lung cancer cells, *SOX21-AS1* negatively modulated the *miR-24-3p*/PIM2 axis to facilitate their proliferation, migration, and invasion. These findings offer a novel idea for future research on treating lung cancer at the molecular level.

## Graphical Abstract

**Figure:**
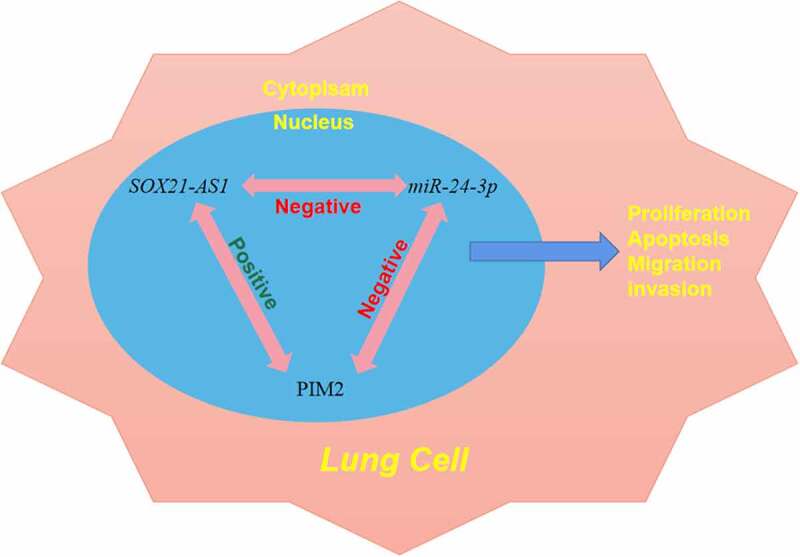

## Introduction

Cancer is the leading cause of death worldwide and has become a serious threat to human health and social development [1]. Presently, deaths caused by lung cancer account for nearly 22.7% of deaths due to cancer, which is the biggest cause of death in China [2,3]. Lung cancer is generally classified as either small cell lung cancer (SCLC) or non-SCLC (NSCLC) based on histopathology, with 80––85% of cases being NSCLC [4,5]. Due to the invasiveness of lung cancer, some patients have local metastasis or infiltration at the initial consultation [6]. The 5-year survival rate of patients with tumor metastasis to other organs or systems is extremely low, but can be more than 40% for patients without tumor metastasis [7]. Studies have indicated that early diagnosis can improve the survival rate of lung cancer patients [8]. Therefore, exploring the molecular mechanism for the treatment of lung cancer has attracted much research interest.

Long non-coding RNAs (lncRNAs) are regarded as RNA fragments greater than 200 nt in length, without the ability to encode proteins [9,10]. Increasing evidence has shown that lncRNAs are ubiquitous in eukaryotes [11]. Recent studies have found that abnormal expression levels of lncRNAs are associated with tumor occurrence, metastasis, prognosis, and diagnosis. They can modulate the expression of protein-coding genes or regulate gene expression transcriptionally or post-transcriptionally, to govern different biological processes, such as proliferation, migration, invasion, apoptosis, and reprogramming of pluripotent stem cells [12–14]. The lncRNA, *SOX21-AS1*, is a newly discovered tumor-related lncRNA. It plays a vital role in a diverse range of cancers, including oral cancer, colorectal cancer, and hepatocellular carcinoma [15–17]. Only two studies have demonstrated an oncogenic role of *SOX21-AS1* in lung cancer [18,19].

MicroRNAs (miRNAs) are RNA transcripts [20]. Increased levels of *miR-24-3p* have been shown to repress Bim to confer tamoxifen resistance in breast cancer cells [21]. TRIM11, which is directly targeted by *miR-24-3p*, accelerates the proliferation and apoptosis of colon cancer cells [22]. Moreover, *miR-24-3p* can interfere with lung adenocarcinoma progression through FGFR3 signaling [23]. PIM2, a downstream target of *miR-24-3p*, has already been shown to exert tumor-promoting effects in different carcinomas. Moreover, a decrease in PIM2 levels inhibits cell proliferation in liver cancer by modulating the cell cycle [24]. PIM2 and STAT3 form a positive feedback loop that regulates epithelial-to-mesenchymal transition in breast cancer [25]. However, the association between these genes has not been explored in the context of lung cancer.

Therefore, the aim of this study was to better understand the molecular mechanism underlying the role of *SOX21-AS1* in lung cancer and to provide new ideas for research on lung cancer treatment. We hypothesized that *SOX21-AS1* regulates the growth and metastasis of lung cancer cells via *miR-24-3p*/PIM2 axis.

## Materials and methods

### Clinical tissues

This study was approved by the Ethics Committee of The First Affiliated Hospital of Soochow University, and informed consent was obtained from all patients. Thirty matched cancerous and noncancerous lung tissue samples were obtained from patients at The First Affiliated Hospital of Soochow University. After surgery, the samples were frozen in liquid nitrogen.

### Bioinformatics prediction

DIANA-LncBase v2 was used to predict the target miRNA of *SOX21-AS1*. TargetScan and miRDB were used to screen for common targets of *miR-24-3p*, and the results were further investigated using Gene Ontology (GO) and Kyoto Encyclopedia of Genes and Genomes (KEGG) pathway analyses.

### Cell culture

The lung cancer cell lines, H125, A549, NCI-H23, HCC827, and NCI-H1299, and normal human lung epithelial BEAS-2B cells were procured from ATCC (Manassas, VA, USA). Cells were grown in RPMI-1640 with 10% fetal bovine serum and 1% double antibody in an incubator at 37°C with 5% CO_2_. When the incubated cells reached 90% confluence, they were digested with trypsin and passaged.

### Reverse transcription quantitative PCR

Total RNA was extracted from tissues and cells using TRIzol reagent (Invitrogen, Carlsbad, CA, USA). The purity and concentration of the RNA samples were determined using a NanoDrop ND-1000 spectrophotometer (Agilent, Santa Clara, CA, USA). The 260 nm/280 nm absorbance ratio of the RNA samples ranged from 1.8 to 2.1. RNA integrity was evaluated by 2% denatured agarose gel electrophoresis. Reverse transcription (RT) was performed using an RT-PCR kit (Promega, Madison, WI, USA). Quantitative (q) PCR was performed using an ABI PRISM 7500 Sequence Detection System (Life Technologies; Thermo Fisher Scientific, Waltham, MA, USA) and a SYBR Green Kit (Takara Biotechnology Co., Kusatsu, Japan). The levels of *SOX21-AS1, miR-24-3p*, and *PIM2* were calculated using the ∆∆Cq method according to a previous study [26] .

### Cell transfection

Vectors containing an *SOX21-AS1* siRNA, *miR-24-3p* inhibitors or mimics, or PIM2, were transfected using Lipofectamine 2000 (Invitrogen), along with the corresponding negative controls (NCs), including an siRNA, miRNA inhibitor, and miRNA mimic NC, miR-NC inhibitor, miR-NC mimics, and pcDNA3.1. Culture medium without any added vector was used as a blank control. Six hours after transfection, the medium was replaced with fresh medium. Gene expression was assessed using RT-qPCR or western blotting to confirm the success of the transfection.

### Colony formation assay

The Colony formation assay was performed as described by Lei et al. [27]. After cell transfection, 2 mL of 0.6% agarose was added to each well of a 6-well plate. Following the solidification of the agarose, the cells were suspended in 0.3% agarose was used to suspend the cells at 37°C, and then 2 mL of the cell suspension was quickly placed on the solidified agarose in the plate (2,000 cells/well). After incubation at 4°C for 10 min, the cells were cultured in a 37°C incubator for 14 d. The number of clones formed was then counted using a microscope (≥ 50 cells were regarded as one clone). Clone formation rate was calculated suing the following formula: (number of clones/number of inoculated cells) × 100.

### MTT assay

MTT assay was conducted according to perious study [28]. Cells (1 × 10^5^) were seeded in 96-well dishes to determine cell viability. At 0, 1, 2, and 3 d after transfection, 20 μL of 3-(4,5-dimethylthiazol-2-yl)-2,5-diphenyltetrazolium bromide (MTT, 5 μg/μL) was added. Dimethyl sulfoxide (150 μL/well) was then added after another 4 h of incubation and the absorbance of each group was measured at 570 nm for each group. The proliferation rates were calculated as the ratios of the absorbance values of the treatment group to the absorbance value of the control group were calculated as the proliferation rate.

### Flow cytometric analysis

Two days after transfection, the cultured cancer cells were digested with trypsin and rinsed with phosphate-buffered saline. The concentration of the resuspended cells was adjusted to 1 × 10^4^ cells/mL. The cells were then incubated with annexin V-FITC and propidium iodide in the dark for 15 min. The rate of apoptosis rate in each group was determined using flow cytometry (FACScan; BD Biosciences, Shanghai, China).

### Transwell migration assay

The transwell migration assay was performed as reported by Omar et al [29]. Transfected cells were cultured for 48 h in Dulbecco’s modified Eagle medium (DMEM) supplemented with 0.1% bovine serum albumin (BSA) at a cell density of 1 × 10^6^ cells/mL. Cells (100 μL) were inoculated into the upper transwell chamber (BD Biosciences), and 500 μL of DMEM containing 15% calf serum was added to the lower chamber. After 1 d of culture at 37°C with 5% CO_2_ at 37°C, cells on the upper layer were removed using a cotton swab. The filter membrane was then fixed with methanol for 5 min and stained with Giemsa stain for 15 min. Cells passing through the membrane were counted in five different fields under a light microscope (Zeiss Axio Observer; Zeiss, Oberkochen, Germany) at a magnification of 100× and the average value was calculated. Migration ability was calculated using the following formula: (1 – the average number of migrated cells in the experimental group/the number of migrated cells vs. those in the control group) × 100%.

### Transwell invasion assay

The transwell invasion assay was performed as reported by Marshall et al. [30]. A Matrigel basement membrane matrix was used to investigate cell invasion. The density of the cells transfected for 48 h was adjusted to 3 × 10^5^ cells/mL and 0.1 mL of the resulting cell suspension was added to each well of the upper transwell chamber containing serum-free medium. One milliliter of complete medium was then added to the lower insert. After 24 h, cells in the lower layer were fixed and stained with crystal violet.

### Luciferase reporter assay

The luciferase reporter assay was conducted according to a previous study [31]. Wild-type (WT) and mutant (MT) fragments of *SOX21-AS1* and *PIM2* were separately amplified by RT-PCR and subcloned into pmirGLO expression vectors (Sangon Biotech, Shanghai, China). The above vectors were then co-transfected into 293 T cells with *miR-24-3p* mimics, miRNA mimics, and mimic NCs using Lipofectamine 2000. A dual-luciferase reporter assay system (TransGene, Beijing, China) was used to detect luciferase activity.

### Tumor xenografts

Tumor xenografts was established as decribed by Lee et al [32]. After successful transfection, a cell suspension of 5 × 10^7^ cells/mL was created via digestion with trypsin, centrifugation, collection, and counting. The cell suspension (0.2 mL) was injected subcutaneously injected into the middle dorsal armpit of nude mice. One week later, tumors appeared *in situ*. Tumor weight and size in each group (20 nude mice) were examined every fourth day. The longest diameter (a) and shortest diameter (b) were measured to calculate tumor volume (V), using the formula, V = πab^2^/6. After 30 d of observation, the nude mice were sacrificed and tumor tissues were immediately collected and weighed. The tumor inhibition rate was then calculated according to the following formula: tumor inhibition rate (%) = (1 – average tumor weight of treatment group/average tumor weight of control group) × 100%. Relative tumor volume (RTV) was calculated according to the formula: RTV = V_t_/V_0_, where V_t_ is the final tumor volume, and V_0_ is the initial tumor volume. Relative tumor proliferation rate (%) was calculated as follows: (the RTV of the treatment group/the RTV of the control group) × 100%.

### Western blotting

The western blotting was conducted according to a previous study [33]. Tissues or cells were treated with RIPA lysis buffer containing protease inhibitors. After centrifugation, the supernatant was heated in a water bath to denature the proteins. Following quantification using the bicinchoninic acid method, proteins were separated by sodium dodecyl sulfate-polyacrylamide gel electrophoresis and then transferred to a polyvinylidene difluoride membrane (Sigma, St Louis, MO, USA). The membranes were probed with primary antibodies against PIM2 (1:1,000; ab129193; Abcam, Cambridge, UK) and GAPDH (1:2,500; ab9485; Abcam) at 4°C for 12 h. Thereafter, the membranes were incubated with a secondary antibody against rabbit IgG (1:1,000; ab190475; Abcam) at room temperature for 1 h. Protein bands were detected using an enhanced chemiluminescence system (Super Signal West Pico Substrate, Thermo Fisher Scientific), followed by quantification using Image J software (National Institutes of Health, Bethesda, MD, USA).

### Statistical analysis

Data from triplicate experiments were analyzed using SPSS software (version 22.0; IBM Corporation, Armonk, NY, USA) and expressed as means ± standard errors of the mean. Differences between two groups or among groups were evaluated using a Student’s t-test or analysis of variance. Statistical significance was set at *P* < 0.05.

## Results

This study aimed to explore the role of *SOX21-AS1* in the occurrence and development of lung cancer cells. We conducted the MTT, colony formation, flow cytometry, and transwell assays to detect the cell proliferation, apoptosis, migration and invasion *in vitro*, and explored the role of SOX21-AS1/miR-24-3p axis in the tumor xenografts *in vivo*.

### SOX21-AS1 *was expressed at high levels in lung cancer*

**Figure 1.**
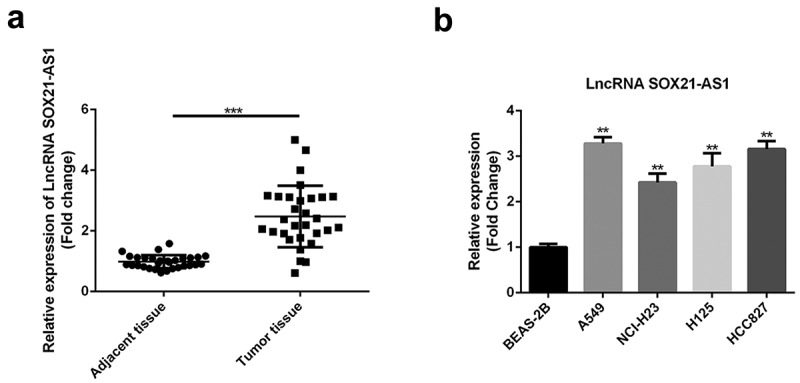
High expression levels of *SOX21-AS1* in lung cancer

The levels of lncRNA *SOX21-AS1* in lung cancer and adjacent tissues were determined using RT-qPCR. The data demonstrated that *SOX21-AS1* expression was markedly higher in cancer tissues than in non-cancer tissues (Figure 1(a)). Moreover, *SOX21-AS1* levels were also examined in cancerous and normal cells. RT-qPCR analysis revealed that *SOX21-AS1* levels were higher in A549, NCI-H23, H125, and HCC827 cells than in BEAS-2B cells (Figure 1(b)). A549 and HCC827 cells showed the highest levels of *SOX21-AS1* among all the cancer cell lines tested. *SOX21-AS1* expression levels were also significantly higher in patients with lung cancer.

(a) *SOX21-AS1* levels in lung cancer and adjacent tissues were determined by RT-qPCR. ***P < 0.001 vs adjacent tissue. (b) The expression of *SOX21-AS1* in normal lung epithelial cells (BEAS-2B) and lung cancer cells (H125, A549, NCI-H23, HCC827, and NCI-H1299) was also analyzed by RT-qPCR. **P < 0.01 vs BEAS-2B cells.

### SOX21-AS1 *targeted* miR-24-3p *in lung cancer*

**Figure 2.**
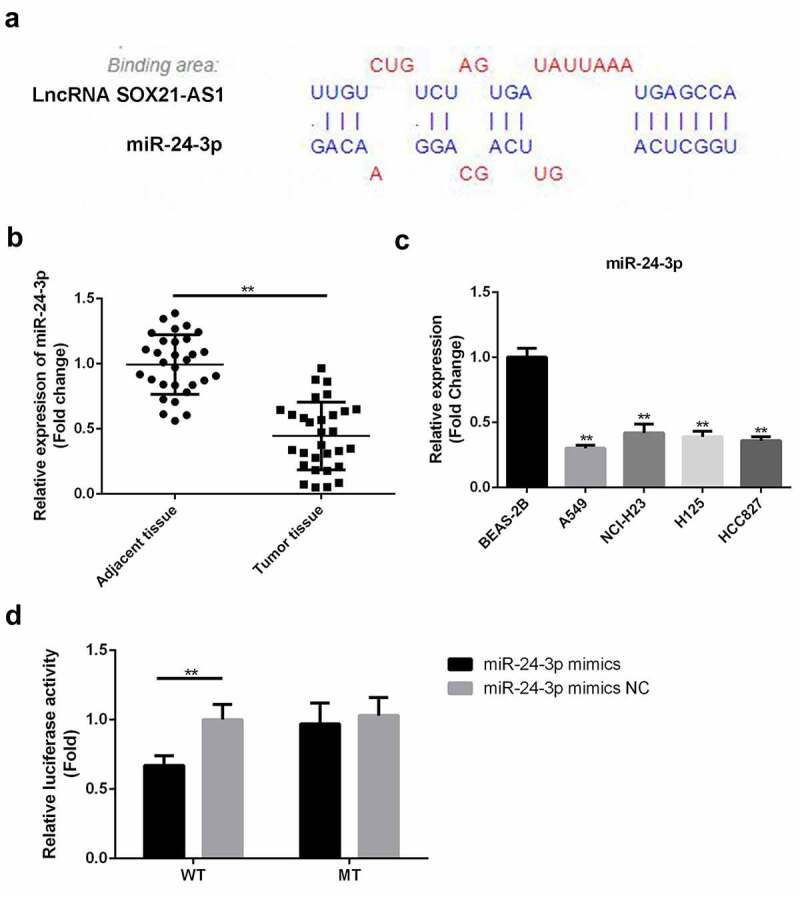
*SOX21-AS1* targeted *miR-24-3p* in lung cancer

Bioinformatics tools revealed that *miR-24-3p* was a potential target of *SOX21-AS1*. The binding area is shown in Figure 2(a). Therefore, we analyzed *miR-24-3p* levels in normal and cancerous tissues. Reduced levels of *miR-24-3p* were observed in cancerous tissues (Figure 2(b)). In addition, *miR-24-3p* levels were markedly decreased in cancerous cells compared to normal BEAS-2B cells (Figure 2(c)). To confirm the binding of *SOX21-AS1* with *miR-24-3p*, a luciferase reporter assay was performed. The luciferase activity of the wild-type *SOX21-AS1* construct was clearly decreased by overexpression of *miR-24-3p* (Figure 2(d)). In summary, we confirmed that *miR-24-3p* bound to *SOX21-AS1*.

(a) The binding site between *SOX21-AS1* and *miR-24-3p* as predicted by DIANA. (b) *miR-24-3p* levels in lung cancer and adjacent tissues were determined by RT-qPCR. **P < 0.01 vs adjacent tissue. (c) *miR-24-3p* expression levels in normal BEAS-2B cells and lung cancer cells were determined by RT-qPCR. **P < 0.01 vs BEAS-2B cells. (d) Luciferase reporter assays were used to assess the binding affinity between *SOX21-AS1* and *miR-24-3p*. **P < 0.01 vs *miR-24-3p* mimic NC.

### miR-24-3p *inhibition reversed the decrease in cell proliferation, apoptosis inhibition, migration, and invasion and the increase in apoptosis induced by* SOX21-AS1 *downregulation*

**Figure 3.**
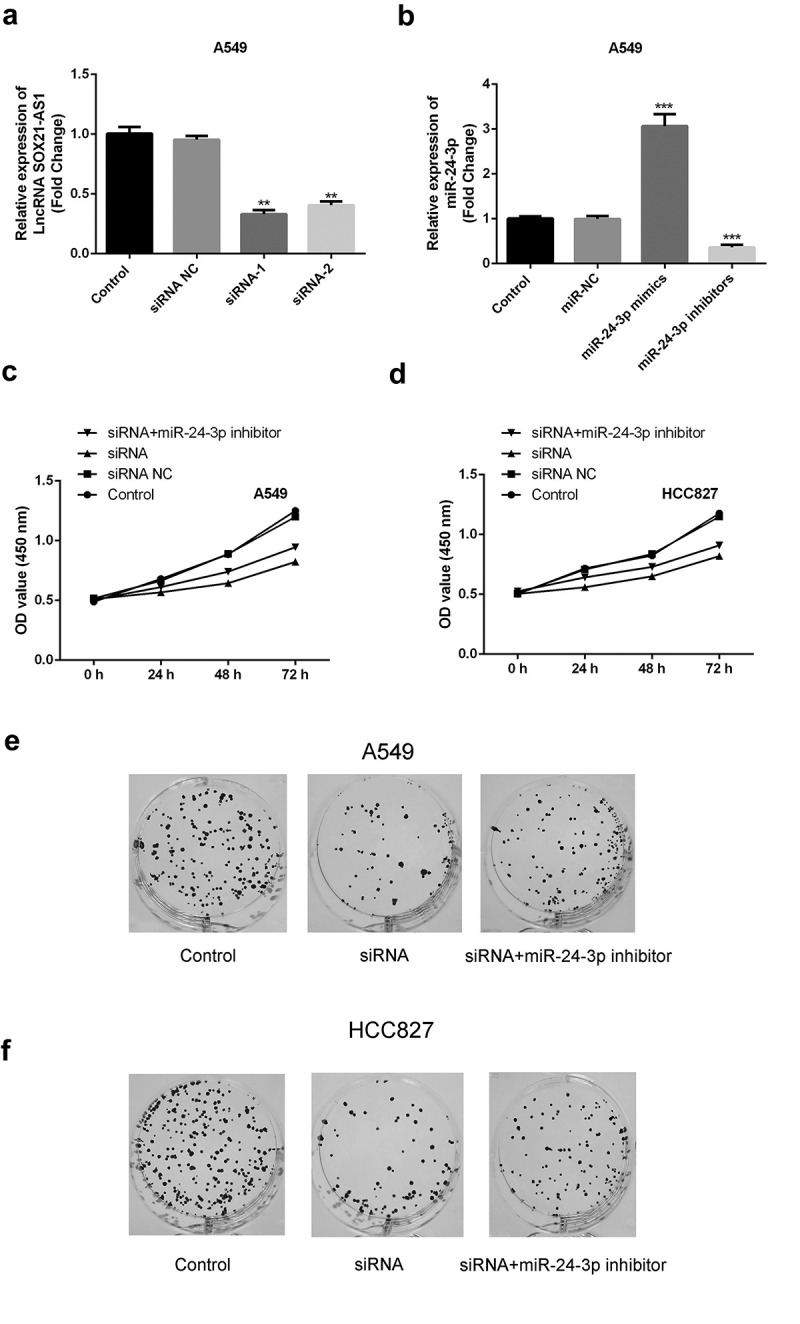
*miR-24-3p* inhibition reversed the decrease in cell proliferation induced by *SOX21-AS1* downregulation

**Figure 4.**
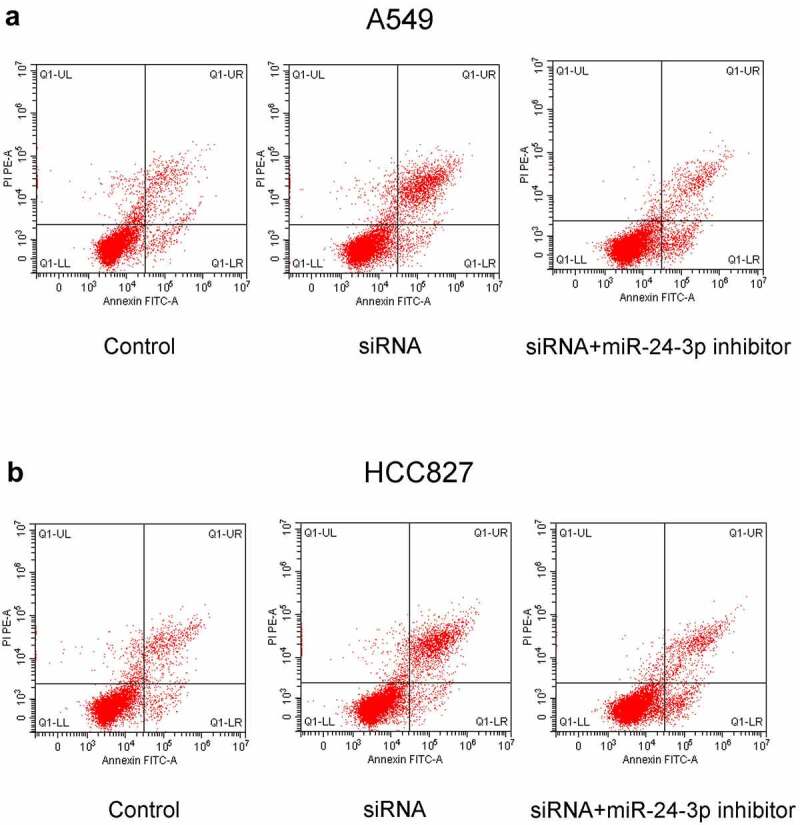
*miR-24-3p* inhibition decreased apoptosis induced by *SOX21-AS1* downregulation

**Figure 5.**
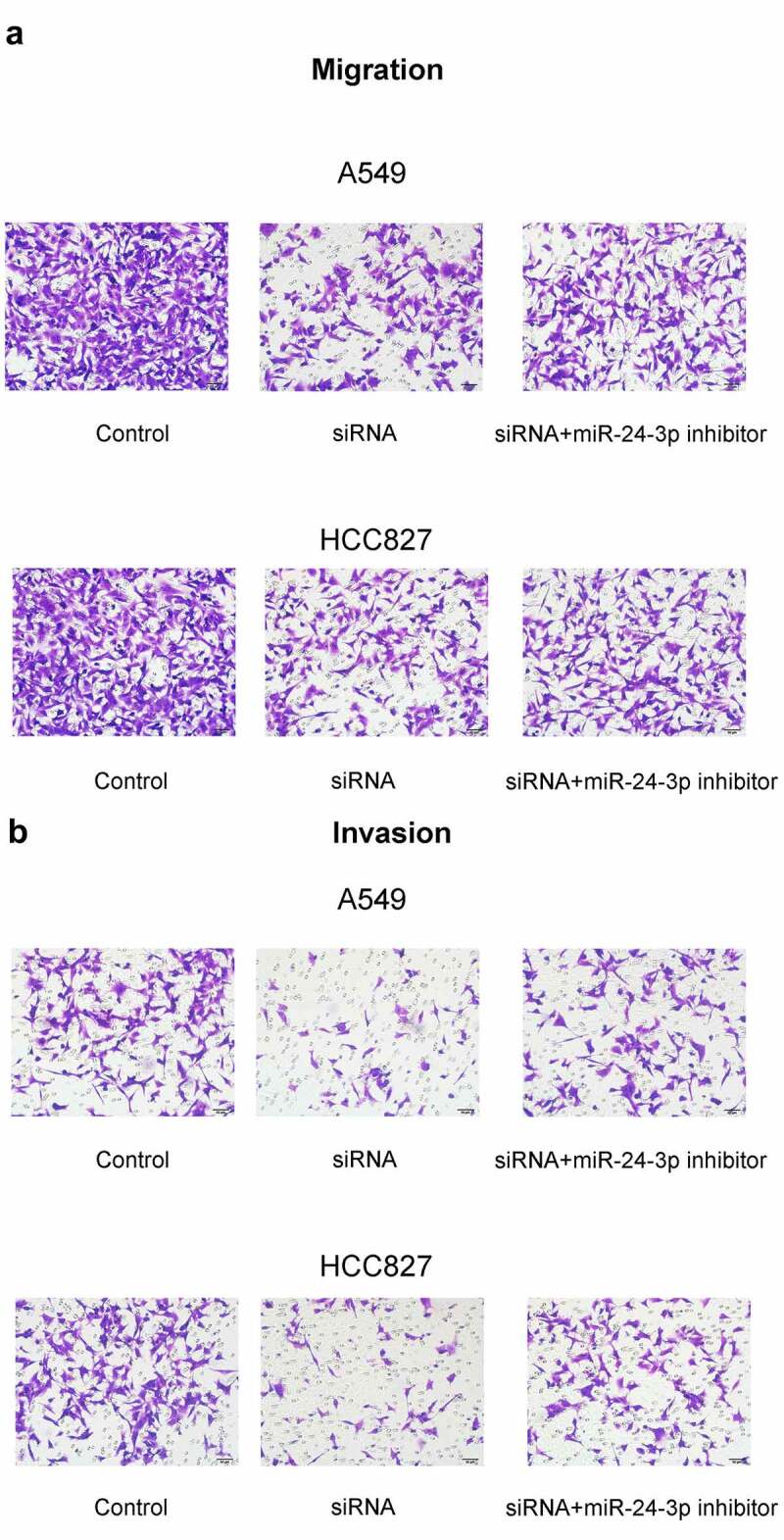
*miR-24-3p* depletion rescued the decrease in cell migration and invasion induced by *SOX21-AS1* downregulation

To determine whether the *SOX21-AS1*/*miR-24-3p* interaction has a role in lung cancer, *SOX21-AS1* expression was reduced, and *miR-24-3p* expression levels were decreased by siRNAs (Figure 3(a,b)). An MTT assay indicated that cell viability was significantly reduced when *SOX21-AS1* expression levels decreased, and this was markedly reversed by *miR-24-3p* depletion (Figure 3(c,d)). Colony formation assays showed similar findings. *SOX21-AS1* inhibition decreased cell proliferation, which was abrogated by the *miR-24-3p* interference (Figure 3(e,f)). In addition, flow cytometric analysis showed that apoptosis was significantly promoted by the silencing of *SOX21-AS1*, and this was rescued upon inhibition of *miR-24-3p* (Figure 4). Transwell experiments showed that both migration and invasion abilities were suppressed by *SOX21-AS1* knockdown, but *miR-24-3p* decreased the inhibition of migration and invasion (Figure 5(a,b)). Thus, the *SOX21-AS1*/*miR-24-3p* axis increased cell proliferation, inhibited apoptosis, and promoted cell migration and invasion.

(a) The knockdown efficiency of siRNAs (siRNA-1 and siRNA-2) targeting *SOX21-AS1* was examined by RT-qPCR. **P < 0.01 vs control. (b) The expression levels and inference efficacy of *miR-24-3p* mimics and an *miR-24-3p* inhibitor were measured by RT-qPCR. ***P < 0.001 vs control. (c-d) An MTT assay was used to evaluate the viability of A549 and HCC827 cells in four different groups. (e-f) Colony formation assays were used to assess the cell proliferation ability of A549 and HCC827 cells.

Apoptosis of A549 (a) and HCC827 (b) cells was analyzed by flow cytometry.

(a-b) The ability of A549 and HCC827 cells to migrate and invade were assessed via transwell experiments.

### miR-24-3p *depletion reversed the decrease in tumor formation induced by* SOX21-AS1 *downregulation*

**Figure 6.**
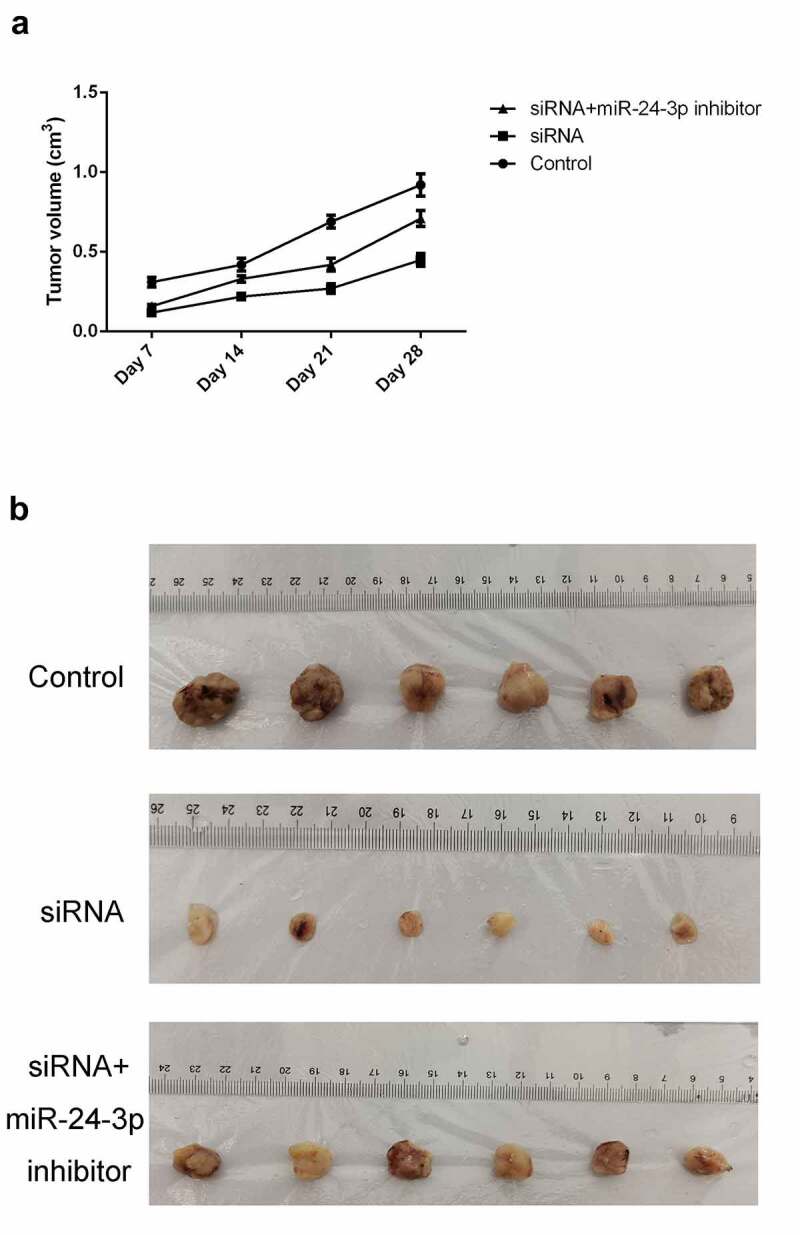
*miR-24-3p* depletion reversed the decrease in tumor formation induced by *SOX21-AS1* downregulation

To confirm the function of the *SOX21-AS1*/*miR-24-3p* axis *in vivo*, animal models were established. Tumor volume markedly decreased upon *SOX21-AS1* depletion, but significantly increased when *miR-24-3p* expression levels were reduced (Figure 6(a)). A similar result was observed for tumor size. Tumors in the siRNA treatment group were significantly smaller than those in the control group, but a moderate tumor size was observed in the siRNA+*miR-24-3p* inhibitor group (Figure 6(b)). Taken together, these results showed that *SOX21-AS1* downregulation inhibited tumor formation, and this effect was reversed upon *miR-24-3p* silencing.

(a) Tumor volumes of mice in the control, siRNA, and siRNA+*miR-24-3p* inhibitor groups. (b) Xenografted tumors from mice in the above three groups are shown.

### miR-24-3p *targeted PIM2, which was overexpressed in lung cancer*

**Figure 7.**
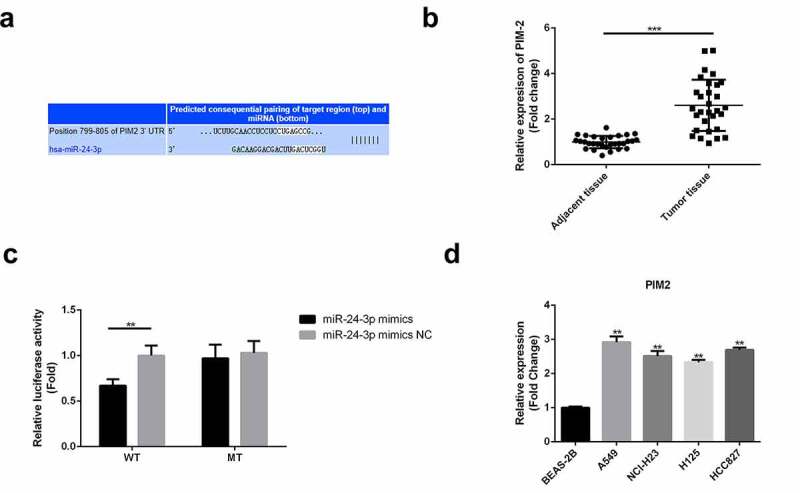
*miR-24-3p* targeted PIM2, which was overexpressed in lung cancer

The downstream mechanism was further investigated. The predicted binding sequence of *miR-24-3p* with *PIM2* is shown in Figure 7(a). *PIM2* levels were markedly higher in lung cancer tissues than in non-tumor tissues (Figure 7(b)). Luciferase reporter assays confirmed that the overexpression of *miR-24-3p* markedly reduced the luciferase activity of the wild-type *PIM2* construct (Figure 7(c)). Moreover, the levels of *PIM2* were higher in cancerous cells than in normal BEAS-2B cells (Figure 7(d)).

(a) The binding sequences of *miR-24-3p* and *PIM2* were predicted using Targetscan. (b) RT-qPCR was used to analyze *PIM2* expression in cancer and non-cancer tissues. ***P < 0.001 vs adjacent tissues. (c) Luciferase reporter assays were used to confirm the targeting of *PIM2* by *miR-24-3p*. **P < 0.01 vs *miR-24-3p* mimic NC. (d) RT-qPCR analysis of *PIM2* expression in cancer and normal cells. **P < 0.01 vs BEAS-2B cells.

### miR-24-3p *over-expression down-regulated the mRNA and protein expression levels of PIM2, inhibited cell proliferation, and promoted the apoptosis*

**Figure 8.**
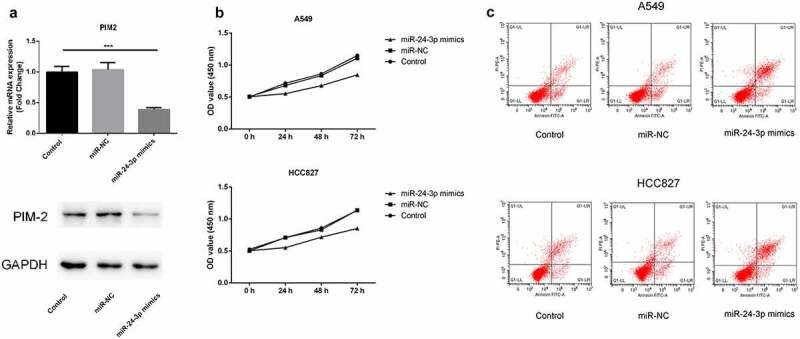
*miR-24-3p* over-expression down-regulated the mRNA and protein expression levels of PIM2, inhibited proliferation, and promoted apoptosis

*miR-24-3p* was overexpressed to explore its impact on PIM2 expression and cellular activity. The mRNA and protein expression levels of PIM2 were downregulated upon overexpression of *miR-24-3p* (Figure 8(a)). MTT assays showed that cell proliferation was markedly repressed by the overexpression of *miR-24-3p* (Figure 8(b)). Flow cytometric analysis showed that *miR-24-3p* overexpression increased apoptosis (Figure 8(c)).

(a) RT-qPCR and western blotting were used to measure mRNA and protein expression levels in A549 cells. ***P < 0.001 vs control. (b) MTT experiments were used to analyze the proliferation of the two cell types. (c) Flow cytometry was used to detect the apoptotic ability of the two tumor cell types.

## Discussion

The levels of *SOX21-AS1* were markedly increased and the levels of *miR-24-3p* were markedly decreased in cancerous tissues and cells. *miR-24-3p* was found to interact with *SOX21-AS1*. The *SOX21-AS1*/*miR-24-3p* axis increased the proliferation, migration, and invasion of A549 and HCC827 cells. Animal models further confirmed the tumor-promoting role of the *SOX21-AS1*/*miR-24-3p* pathway. PIM2, which showed elevated levels in lung cancer, was the target of *miR-24-3p*. The targeting of PIM2 by *miR-24-3p* disrupted the cellular activities of lung cancer cells.

As a newly discovered lncRNA, *SOX21-AS1* has been shown to increase tumor progression. *SOX21-AS1* facilitates the progression of cervical cancer by targeting the *miR-7*/VDAC1 pathway [34]. *SOX21-AS1* also positively regulates the progression of triple-negative breast cancer via the *miR-520a-5p*/ORMDL3 axis [35]. *SOX21-AS1* increases the proliferation and invasion properties of gliomas by increasing PAK7 levels through the absorption of *miR-144-3p* [36]. Furthermore, two studies have demonstrated the oncogenic function of *SOX21-AS1* in lung cancer. *SOX21-AS1* predicts prognosis and potentiates proliferation in lung adenocarcinoma, and its silencing represses migration and invasion by regulating GATA6, which decreases the levels of TSPAN8 [18,19]. In this study, *SOX21-AS1* was also highly expressed in both lung cancer tissues and cells, which is consistent with the findings of previous studies. The results in this study and others implied that *SOX21-AS1* is a oncogenic lncRNA in lung cancer.

To further elucidate the novel molecular mechanism of *SOX21-AS1* in lung cancer, DIANA-LncBase v2 was used to identify the binding sequence between *SOX21-AS1* and *miR-24-3p*. Several studies have demonstrated the role of *miR-24-3p* as a tumor suppressor. For example, silencing the lncRNA, *CCAT1*, decreases paclitaxel resistance in prostate cancer by upregulating *miR-24-3p* and decreasing FSCN1 levels [37,38]. *miR-24-3p* inhibits the progression of pancreatic ductal adenocarcinoma by decreasing LAMB3 expression levels [39]. In addition, *miR-24-3p* can inhibit lung adenocarcinoma progression by mediating FGFR3 signaling [23]. In the present study, we observed that *miR-24-3p* levels were markedly lower in lung cancer tissues and cells than in normal tissues and cells. Moreover, *miR-24-3p* interacted with *SOX21-AS1*. Functional assays confirmed that *miR-24-3p* interference abrogated the inhibition of SOX21-AS1 downregulation on cell proliferation, migration, and invasion, and the promotion of cell apoptosis induced by the downregulation of *SOX21-AS1*. These results indicated that *SOX21-AS1* induces the apoptosis and inhibits the growth of the lung cancer cells via negatively regulating the *miR-24-3p* expression.

Based on these results, an in-depth investigation of the associated mechanism was performed. Using Targetscan, miRDB, GO, and KEGG, *PIM2* was predicted to be the target of *miR-24-3p*. According to previous reports, PIM2 promotes the progression of a diverse range of cancers. In liver cancer, the inhibition of PIM2 decreases cell proliferation by modulating the cell cycle [24]. PIM2 facilitates HCC progression via NF-κB signaling [39]. PIM2 also interacts with tristetraprolin, thereby promoting breast cancer progression [40]. Consistent with the results from previous studies, lung cancer tissues and cells were found to have relatively high expression levels of PIM2. Further, functional experiments demonstrated that cell proliferation was inhibited and apoptosis was promoted by the upregulation of *miR-24-3p*. We confirmed that *SOX21-AS1* can interact with *miR-24-3p* through competitive endogenous RNA mechanism, thereby affecting the expression of PIM2 and relieving the lung cancer development.

## Conclusion

In conclusion, this study showed that *SOX21-AS1* negatively modulated the *miR-24-3p*/PIM2 axis to potentiate the proliferation, migration, and invasion capabilities of lung cancer cells. This may offer a novel molecular avenue for research into molecular lung cancer therapies.

## Data Availability

The authors confirm that the data supporting the findings of this study are available within the article.

## Highlights

1. SOX21-AS1 is a newly discovered oncogenic lncRNA

2. SOX21-AS1 is up-regulated in lung cancer

3. SOX21-AS1 negatively modulated the miR-24-3p/PIM2 axis
